# Supplementary material for: System identifiability in a time-evolving agent-based model
Source: PLoS One. 2024 Jan 25;19(1):e0290821. doi: 10.1371/journal.pone.0290821 (PMC10810497; doi:10.1371/journal.pone.0290821)
Supplement: S1 File — Pseudo-codes for algorithms used for computation in this paper. (DOCX) [file pone.0290821.s001.docx]

# Supplementary Materials

## Pseudo codes for the algorithms used in the paper:

### Algorithm 1 - Daily Ensemble Progression of Colonization Status:

$$\left[ {\hat{\boldsymbol{C}}}^{\boldsymbol{d+1}}\boldsymbol{,}{\bar{\boldsymbol{O}}}^{\boldsymbol{d+1}} \right]\boldsymbol{=}\boldsymbol{f}_{\boldsymbol{1}}\left( \boldsymbol{G}^{\boldsymbol{d}}\boldsymbol{,}\hat{\boldsymbol{\Theta}}\boldsymbol{,}{\hat{\boldsymbol{C}}}^{\boldsymbol{d}} \right)$$

**Input:**

1. $G^{d}(p_{first}^{d},k^{d},p_{w}^{d},N_{w})$ – Network contact details for day d.
   1. $p_{first}^{d}$ – patients newly admitted on day d.
   2. $k_{i}^{d}$ – number of wards a patient visit on day d.
   3. $p_{w}^{d}$ - all patients in ward $w$ on day d
   4. $N_{w}$ – the size of each ward.
2. $\hat{\Theta}$ – ensemble of parameters. $\hat{\Theta}_{e,j}$ is the $j_{th}$ parameter of ensemble member $e$. ($E\times4$ matrix).
3. $\hat{C}^{d}$ – ensemble of colonization states for all patients. $\hat{C}_{n,e}^{d}=1/0$ if the $n_{th}$ patient of ensemble member $e$ is colonized/uncolonized, respectively ($N_{tot}\times E$ matrix).

**Progression:**

For each ensemble member $e$:

1. Create probability vector: $\bar{C}^{*}=\bar{C}_{e}^{d}$
2. Decay from previous day: $\bar{C}^{*}=\left( 1-\alpha_{e} \right)\bar{C}^{*}$
3. For each ward, $w$:
   1. Calculate the infection force of the ward:
      $F_{w,e}^{d}=\sum_{j\in p_{w}} \frac{1}{k_{j}^{t}}\hat{C}_{j,e}^{d}$
   2. For each patient $i$ in ward $w$ ($i\in p_{w}^{d}$), who is susceptible on day, add the probability to be colonized while in the ward: $C_{i}^{*}=C_{i}^{*}+\left( 1-C_{i,e}^{d} \right)\frac{1}{k_{i}^{d}}\frac{\beta_{e}}{N_{w}}F_{w,e}^{d}$
4. For each patient for whom this is the first hospitalization day ($i\in p_{first}^{d}$), set importation probability:

$$C_{i}^{*}=\gamma$$

5. Draw a random number, $r_{i,e}\sim uniform\left( \left[ 0,1 \right] \right)$ and set colonization status for the next day such that:

$$C_{i,e}^{d+1}=\left\{ \begin{matrix} 0 & C_{i}^{*}<r_{i} \\ 1 & C_{i}^{*}\geq r_{i} \end{matrix} \right.$$

6. Draw a random number, $r_{i}\sim uniform\left( \left[ 0,1 \right] \right)$ and set observation status of the next day such that:

$$\hat{O}_{i,e}=\left\{ \begin{matrix} 0 & C_{i,e}^{d+1}\cdot\rho<r_{i}' \\ 1 & C_{i,e}^{d+1}\cdot\rho\geq r_{i}^{'} \end{matrix} \right.$$

**Return:**

1. $C^{d+1}$ – the colonization status for day $d+1$ ($N_{tot}\times1$ vector).
2. $\bar{O}^{d+1}$ – number of positive results observed on day $d+1$. $\bar{O}_{e}^{d+1}=\sum_{i=1}^{N_{tot}} \hat{O}_{i,e}$.

### Algorithm 2 - Iterated Ensemble Adjustment Kalman Filter:

$$\left[ \hat{\Theta} \right]=f_{2}\left[ d_{step},E, N_{i},\bar{O}_{r}, \hat{R}, f_{1},p_{o} \right]$$

**Input:**

1. $d_{step}$ – number of days in assimilation period.
2. $E$ - Number of ensemble members.
3. $N_{i}$ – number of iterations
4. $\bar{O}_{r}$ – the number of observed positive results in each assimilation period; a vector of $1\times T$
5. $\hat{R}$ – initial range of parameters $\left( \beta,\gamma,\alpha,\rho\right)$, $\hat{R}_{1,j}$ and $\hat{R}_{2,j}$ are the lower and upper limits of the $j_{th}$ parameter, respectively. ($2\times4$ matrix)

$\left[ \hat{C}^{d+1},\bar{O}^{d+1} \right]=f_{1}\left( G^{d},\hat{\Theta},\hat{C}^{d} \right)$

7. $p_{0}$ – probability of colonization among patients already in hospital on day 0.

**Progression:**

1. For each parameter $j$, for each ensemble member $e$, draw values from the range:

$$\hat{\Theta}_{e,j}\sim uniform\left( \left[ \hat{R}_{1,j},\hat{R}_{2,j} \right] \right)$$

2. For $i=1:N_{i}$ iterations:
   1. Initialize colonization state: For each patient, $i$, and each ensemble member $e$, draw a random number, $r_{e,i}\sim uniform\left( \left[ 0,1 \right] \right)$ and set the colonization state:
      $\hat{C}^{1}\left( e,i \right)=\left\{ \begin{matrix} 0 & p_{0}<r_{e,i} \\ 1 & p_{0}\geq r_{e,i} \end{matrix} \right.$
   2. Initialize the prior of the ensemble parameters by posterior of previous iteration:
      $\hat{\Theta}_{e,j}^{0}=\hat{\Theta}_{e,j}$
   3. For $t=1:T$ timesteps:
      1. For each day in timestep :
         1. Progress colonization: $\left[ \hat{C}^{d+1},\bar{O}^{d+1} \right]=f_{1}\left( G^{d},\hat{\Theta},\hat{C}^{d} \right)$
         2. Sum observations: $\bar{O}_{t}=\bar{O}_{t}+\bar{O}_{d}$
      2. Use the Ensemble Adjustment Kalman Filter to update the parameter ensemble (Algorithm 3): $\left[ \hat{\Theta}^{t} \right]=f_{3}\left[ \bar{O}_{t}, O_{r}^{t-1},\hat{\Theta}^{t},\sigma_{obs},\hat{R} \right]$
      3. If the variance of the parameter ensemble decreases too rapidly, add noise using the Jittering algorithm (Algorithm 4):
         $\left[ \hat{\Theta}^{t} \right]=f_{4}\left( v_{w},\hat{\Theta}^{t} ,\hat{R} \right)$
   4. For each ensemble member, calculate the parameter ensemble $\hat{\Theta}$ as the average over iteration periods:

$$\hat{\Theta}_{e,j}=\frac{1}{T}\sum_{t=1}^{T} \hat{\Theta}_{e,j}^{t}$$

**Return:** $\hat{\Theta}$

### Algorithm 3 - Update Variables:

$$\left[ \hat{\Theta} \right]=f_{3}\left[ \bar{O}_{t}, O_{r}^{t},\hat{\Theta},\sigma_{obs},\hat{R} \right]$$

**Input:**

1. $\bar{O}_{t}$ – number of observed positive tests during the assimilation period for each ensemble member.
2. $O_{r}^{t}$ – number of observed positive tests during the assimilation period in the real trajectory.
3. $\hat{\Theta}$ – ensemble of parameters. $\hat{\Theta}_{e,j}$ is the $j_{th}$ parameter of ensemble member $e$. ($E\times4$ matrix). Each column, the ensemble average, is denoted by $\bar{\theta}_{j}$.
4. $\sigma_{obs}$ – the expected standard deviation of the observations.
5. $\hat{R}$ – range of parameters $\left( \beta,\gamma,\alpha,\rho\right)$, $\hat{R}_{1,j}$ and $\hat{R}_{2,j}$ are the lower and upper limits of the $j_{th}$ parameter, respectively ($2\times4$ matrix)

**Progression:**

1. Observation variance: $v_{obs}=1+\left( \sigma_{obs}\cdot O_{r}^{t} \right)^{2}$
2. Posterior variance: $v_{post}=\frac{var\left( \bar{O}_{t} \right)\cdot v_{obs}}{var\left( \bar{O}_{t} \right)+v_{obs}}$
3. Posterior mean: $\mu_{post}=v_{post}\left( \frac{mean\left( \bar{O}_{t} \right)}{var\left( \bar{O}_{t} \right)}+\frac{O_{r}^{t}}{v_{obs}} \right)$
4. Kalman gain: $d\bar{y}=\mu_{post}+\sqrt{\frac{v_{obs}}{v_{obs}+var\left( \bar{O}_{t} \right)}}\left( \bar{O}_{t}-mean\left( \bar{O}_{t} \right) \right)-\bar{O}_{t}$
5. For each parameter $j$
   1. Kalman gain for each parameter $d\bar{\theta}_{j}=d\bar{y}\cdot\frac{cov\left( \bar{\theta}_{j},\bar{O}_{t} \right)}{var\left( \bar{O}_{t} \right)}$
   2. Update ensemble: $\bar{\theta}_{j}=\hat{\theta}_{\theta}+d\bar{\theta}_{j}$
   3. Check if all ensemble members are within parameter range. For each ensemble member below lower bound – set to lower bound. For each ensemble member above upper bound – set to upper bound.

**Return:**

$\hat{\Theta}$ – updated ensemble of parameters.

### Algorithm 4 - Jittering to correct variance:

$$\left[ \hat{\boldsymbol{\Theta}} \right]\boldsymbol{=}\boldsymbol{f}_{\boldsymbol{4}}\left( \boldsymbol{v}_{\boldsymbol{w}}\boldsymbol{,}\hat{\boldsymbol{\Theta}}\mathbf{,}\hat{\mathbf{R}} \right)$$

**Input:**

1. $v_{w}$ – minimum desired ensemble variance
2. $\hat{\Theta}$ – ensemble of parameters. $\hat{\Theta}_{e,j}$ is the $j_{th}$ parameter of ensemble member $e$. Each column is denoted by $\bar{\theta}_{j}$. ($E\times4$ matrix).
3. $\hat{R}$ – range of parameters $\left( \beta,\gamma,\alpha,\rho\right)$, $\hat{R}_{1,j}$ and $\hat{R}_{2,j}$ are the lower and upper limits of the $j_{th}$ parameter, respectively. ($2\times4$ matrix)

**Progression:**

for each variable $j=1:4$

If: $var\left( \bar{\theta}_{j} \right)>v_{w}\cdot\left( R_{2,j}-R_{1,j} \right)$ :

1. Leave $\bar{\theta}_{j}$ unchanged.

Else:

$v_{n}=v_{w}\cdot\left( \hat{R}_{2,j}-\hat{R}_{1,j} \right)-var\left( \bar{\theta}_{j} \right)$

1. Draw $E\times4$ matrix of random numbers from a normal distribution $\eta_{e,j}\mathcal{\sim N}\left( 0, v_{n} \right)$

$\bar{\theta}=\bar{\theta}+\bar{\eta}$

**Return**: $\hat{\Theta}$

### Algorithm 5 - Calculating the likelihood of parameter set $\boldsymbol{\theta}_{\boldsymbol{i}}$ given trajectory ${\bar{\boldsymbol{O}}}_{\boldsymbol{j}}$:

$$\Pr\left( \bar{O}_{j} | \theta\right)=f_{5}\left( \bar{O}_{r},\theta,d_{step},E, p_{0} \right)$$

**Input:**

1. $\bar{O}_{r}$ – the number of observed positive results in each assimilation period; a vector of length $T$
2. $\theta$ – vector of parameters.
3. $d_{step}$ – number of days in each assimilation period.
4. $E$ - Number of ensemble members.
5. Daily progression algorithm: $\left[ \hat{C}^{d+1},\bar{O}^{d+1} \right]=f_{1}\left( G^{d},\hat{\Theta},\hat{C}^{d} \right)$
6. $p_{0}$ – probability of colonization among patients already in hospital on the day of model initialization.

**Progression:**

1. Initialize colonization state: For each patient, $i$, and each ensemble member $e$, draw a random number, $r_{e,i}\sim uniform\left( \left[ 0,1 \right] \right)$ and set the colonization state:
   $\hat{C}^{1}\left( e,i \right)=\left\{ \begin{matrix} 0 & p_{0}<r_{e,i} \\ 1 & p_{0}\geq r_{e,i} \end{matrix} \right.$
   1. For $t=1:T$ timesteps:
      1. For each day in timestep :
         1. Progress colonization: $\left[ \hat{C}^{d+1},\bar{O}^{d+1} \right]=f_{1}\left( G^{d},\hat{\Theta},\hat{C}^{d} \right)$
         2. Sum observations: $\bar{O}_{t}=\bar{O}_{t}+\bar{O}_{d}$
      2. Calculate mean and standard deviation of $\bar{O}_{t}$:
         $\mu_{\theta}^{t}=mean\left( \bar{O}_{t} \right) ; \sigma_{\theta}^{t}=std\left( \bar{O}_{t} \right)$
      3. Calculate probability to observe $\bar{O}_{r}^{t}$ using a discrete normal distribution with mean ${\mu_{\theta}}_{i}^{t}$ and standard deviation $\sigma_{\theta_{i}}^{t}$:
         $\Pr\left( \bar{O}^{t}=\bar{O}_{r}^{t}|\theta\right)=\frac{1}{2}\left[ \mathrm{erf} \left( \frac{\bar{O}_{r}^{t}-\mu_{\theta}^{t}+0.5}{\sqrt{2}\sigma_{\theta}^{t}} \right)-\mathrm{erf} \left( \frac{\bar{O}_{r}^{t}-\mu_{\theta}^{t}-0.5}{\sqrt{2}\sigma_{\theta}^{t}} \right) \right]$
   2. Calculate probability of entire trajectory $\bar{O}_{r}$

$$\Pr\left( \bar{O}_{j} | \theta\right)=\prod_{t=1}^{T} \Pr\left( \bar{O}^{t}=\bar{O}_{r}^{t}|\theta\right)$$

**Return:** $\Pr\left( \bar{O}_{j} | \theta\right)$
