# Supplementary material for: System identifiability in a time-evolving agent-based model
Source: PLoS One. 2024 Jan 25;19(1):e0290821. doi: 10.1371/journal.pone.0290821 (PMC10810497; doi:10.1371/journal.pone.0290821)
Supplement: Attachment — Submitted filename: Reply to rejection letter.docx [file pone.0290821.s002.docx]

To: PLOS One editor,

Attached below is the letter of rejection our paper received from PLOS computational biology on 09/12/2022.

As can be seen, the sole objection brought forth by the editors was that the issue of parameter identifiability is not novel. While we can not dispute this argument, we still feel there is merit in our manuscript for two reasons:

First, our paper is focused specifically on stochastic models such as agent-based models. Unlike deterministic models such as compartmental models, the issue of parameter identifiability in stochastic model is discussed less in the literature and is not as well defined (as we discuss in our introduction). Many of the suggested analytic tools and solutions for non-identifiable deterministic models are not suitable for stochastic models. Here we offer a steppingstone, hopefully part of a larger scientific discussion, on the path to better define, identify and analyze identifiability in stochastic infectious disease models.

Second, while it is indeed true that the issue of non-identifiability is known to the scientific community, it unfortunately remains an issue that is often overlooked. In our manuscript, we show how an inference that converges reliably and yields parameters that reproduce observation well, cannot be claimed identifiable. Many studies using stochastic and deterministic models neglect proper examination of identifiability and there is a good chance that many accepted claims would not withstand rigorous scrutiny. We, therefore, believe that it is an issue worthy of consideration.

All the best,

Tal Robin.

======================================================================================================================================

Dear Dr. Robin:

Thank you very much for submitting your manuscript "System Identifiability in a time-evolving agent-based model" (PCOMPBIOL-D-22-01214) for review by PLOS Computational Biology.

The manuscript has been reviewed and discussed internally by members of the editorial board. Unfortunately, there is a consensus that the manuscript would not do well during an external peer review process, and we will be unable to consider the manuscript further at PLOS Computational Biology.

The issues highlighted by the central model in this manuscript, parameter identifiability due to data sparseness and/or model complexity, are well-known in the computational modeling community (see, e.g., recent reviews by Wieland et al and Villaverde et al.). The article does not claim present any substantial technical advances in this area. In this sense it seems almost more appropriate as a perspective or a tutorial rather than a primary research article in this field. Perhaps it would be more appropriate for a more specialized journal in the field of public health or disease modeling?


While we cannot consider your manuscript further for publication in PLOS Computational Biology, we would like to offer you the option to transfer your submission to PLOS ONE https://www.editorialmanager.com/PONE/
.

If you DO wish to transfer your submission, please click this link:

********

If you do NOT wish to transfer your submission, please click this link to decline:

********

Please note, all PLOS journals are editorially independent and vary in submission requirements.

Should you choose to transfer, your submission details and manuscript files will be transferred automatically, and you will receive a confirmation email within 24 hours. Once transferred, your submission will be returned to you so you can check over your record before completing the submission. You may be asked to provide additional information. If you have any questions, please contact the editorial office of PLOS ONE https://www.editorialmanager.com/PONE/


Thank you once again for your support of PLOS and of open-access publishing. We hope that you will consider PLOS Computational Biology for other submissions in the future.

Sincerely,

James R. Faeder
Academic Editor
PLOS Computational Biology

Thomas Leitner
Section Editor
PLOS Computational Biology


References

1. Villaverde, A. F., Pathirana, D., Fröhlich, F., Hasenauer, J. & Banga, J. R. A protocol for dynamic model calibration. Briefings in Bioinformatics 23x, 1–19 (2022).

2. Wieland, F.-G., Hauber, A. L., Rosenblatt, M., Tönsing, C. & Timmer, J. On structural and practical identifiability. Current Opinion in Systems Biology 25, 60–69 (2021).

__________________________________________________
In compliance with data protection regulations, you may request that we remove your personal registration details at any time. (Use the following URL: https://www.editorialmanager.com/pcompbiol/login.asp?a=r). Please contact the publication office if you have any questions.
