# Supplementary material for: System identifiability in a time-evolving agent-based model
Source: PLoS One. 2024 Jan 25;19(1):e0290821. doi: 10.1371/journal.pone.0290821 (PMC10810497; doi:10.1371/journal.pone.0290821)
Supplement: Attachment — Submitted filename: Reply to reviewers.docx [file pone.0290821.s003.docx]

Dear editor,

Below are the comments from reviewers and our replies.

We hope this satisfies all concerns and will be happy to make additional changes if those are deemed necessary.

We addressed all editorial comments in the submission and made a suppository of our data and algorithms that reproduce all figures:

<https://github.com/TalRobin/ABM_Identifiability>

All the best,

Tal Robin.

## Reviewer comments:

### Reviewer 1:

1. The abstract talks about an approach to discern whether model parameters have been properly identified and about a suggestion for data manipulation. After this, I continued reading, looking for some general description of the procedures and the ambit of application, which I could not find.

Reply:

We thank the reviewer for this comment. In our results (Lines 174-181 and Figure 5 in previous version) we show that the inference algorithm is unreliable in retrieving the correct parameters. However, we agree this message was not brought forth strongly enough and therefore made the following changes to the revised manuscript. Please see revision in lines 244-253 in revised manuscript.

In the discussion (Lines 215-226) we provide suggestions to improve identifiability. We are currently working on implementation and examination of these suggested approaches. This work is involved, and it is our intention to publish these results separately.

I feel that the term identifiability is used to refer to so many concepts that, at the end, it is difficult to keep up. For example, It is said that one of the issues that can affect the identifiability of a system is the identifiability of the model, which I understand relates to structural identifiability, and when talking about the data the term practical identifiability, which has also been mentioned, is not used.

Reply: The classification of identifiability into structural and practical categories, while established for deterministic models is less well defined for stochastic models where structural and practical can overlap; Instead, we identify 4 sources affecting identifiability in evaluating our system, as discussed on Lines 263-285. We feel this classification captures the processes at play for this system and have clarified this in the revised manuscript.

2. (Line 126) "we will assume knowledge of two parameters, α and α" the same parameter is mentioned.

Reply: This typo has been fixed, thanks for bringing it to our attention.

3. (Line 138) The reference to Figure 1 is wrong.

Reply: Mislabeling fixed, thanks for bringing it to our attention.

4. (Line 194) The term "demonstrated" might lead to the thought that a demonstration has been developed rather than an interpretation of results.

Reply: We changed the wording from “demonstrated” to “show”. Thank you for your remark.

5. (Lines 300 and 301) The subtraction is divided into two lines which seems a bit confusing.

Reply: We agree that the alignment of the equation was confusing, as well was the phrasing of the entire paragraph. Please see lines 157-168 in the revised manuscript.

### Reviewer 2:

1. I'm not sure what the goal of the study is. Is the goal of the study to "systematically evaluate" the issue of identifiability or to show a case study where this issue matters? From the title, abstract, and introduction, I got the sense that the point was to evaluate the issue. But, from the methods and results, I got the sense that this is a case study where the authors show the implications of unidentifiable parameters in an ABM calibrated to MRSA data from NYPH. From lines 215 to 225, the authors themselves outlined some solutions to make the parameters identifiable, but did not explore those options in their methods. Another potential solution to this issue would be to provide priors to the importation and transmission rate. How would that change the results?

Reply: In our paper we are aiming to illustrate a broader concept using a simple case study: here a stochastic ABM informed by time-varying real-world data. However, the issues that we are discussing are not unique to the example we provide. By using the agent-based model of MRSA colonization in NY hospitals, we demonstrate the broader issue of how stochasticity and model structure can conspire to undermine identifiability.

For this publication we limit the scope for two reasons. First, we are currently working on the implementation of these approaches and the scope of that discussion is larger than this paper and will be included in a future publication. Additionally, while we think that these suggestions are relatively general and are aimed at inspiring people to think of methods appropriate to their systems, the application to our model is too involved in the specifics of our system.

2. I think the experiments are not necessarily enough to support the authors' conclusions. In my perception, it is not as important to determine whether an interplay of parameters might lead to parameters being unidentifiable as much as what are the requirements for those parameters to be identifiable. I suggest that the authors design additional experiments to determine these regions.

Reply: Please see reply to the following question.

3. The authors did not provide enough motivation about the issue with those parameters being unidentifiable. For instance, what were the issues with those parameters being unidentifiable? Did the trajectories diverge in predictions? If the goal of a model is to project the disease dynamics, would that matter? As the authors mentioned, one issue could be the implementation of interventions targeted to a specific parameter. I encourage the authors to perform such simulations to determine the impact of unidentifiable parameters on public health interventions.

Reply: Thank you for your remark. Figure 4 and the text supporting it were meant to stress the importance of being able to distinguish between different parameter sets in the equi-likelihood ridge. There, we show that though the parameters produce indistinguishable predictions for the observed variable (positive test number) and fail to predict hidden variables, such as total number of colonization and the ratio between nosocomial and community colonization cases However, we think that looking at the effect these discrepancies have on intervention would really bring home the message, and we thank you for suggesting this. We have added an example of an intervention (for example, intensified surface cleaning) represented as a proportional change in the nosocomial infection rate. Please see figure 5 in the revised manuscript for more details.

4. The structure of the manuscript could be better organized. Several sections in the results should be part of the methods.

Reply: We agree that the structure of the manuscript was not ideal. We have now moved the methods before the results and moved the algorithms to the supplementary.

5. I think there is a lot of information about the algorithms that could probably go to the supplement.

Reply: Indeed, we agree. All algorithms were moved to the SI and some of the details about the calculations was removed all together.

6. In the introduction, the authors claim that parameter identifiability is often disregarded in modeling studies, but they don't show examples of that.

Reply: We find that it is a custom in our field and others that inference is conducted to only show convergence of the inference algorithm and to show that observations were reasonably reproduced (for example, many researchers use the “Pomp” package which produce parameter estimates but do not examine system identifiability). Here, we prefer to avoid pointing fingers to any specific publications and hope that people will take this message to heart regardless.

7. In line 81 the authors mention that ABMs are increasingly used in real-world applications but don't mention the literature.

Reply: Thank you for bringing this to our attention, we added some references.

8. Line 82. Is this study considered a systematic evaluation?

Reply: We do not consider this work a systematic evaluation on its own; however, we do consider it the first step of a larger evaluation of identifiability issues specific to agent-based models and other intrinsically stochastic models. Our analysis contributes by offering a broader classification of the issues plaguing identifiability in such systems and by providing an example that is simple, addresses several issues, and is easily generalizable.

9. Lines 122-124. The authors give a lot of unnecessary descriptions of stochastic models.

Reply: While we agree that some readers might find this description trivial, we included it both because other readers might not and mostly to build the narrative of how identifiability is compromised by the nature of stochastic models.

10. Time tense: sometimes future sometimes past. A bit confusing.

Reply: We thank the reviewer for this editorial remark. We have transformed the relevant sentences to the past tense.

11. Line 140. What does "free simulation" mean?

Reply: We used the term “free simulation” to describe a simulation in which parameters remain constant throughout the run time, to be distinguished from simulations for which parameters are adjusted due to the inference algorithm. In the revision, we decided to remove the word “free” and simply use ‘simulation’.

12. Lines 167-168. Why did the authors not perform scenarios of different targeted interventions?

Reply: While we initially avoided dealing with the issues of intervention due to the complexity in aptly describing its effects in theoretical models, the tried to comply with the reviewer suggestion and provide a schematic representation of an intervention (such that will reduce nosocomial transmission proportional to its native rate). This turned out to be a very elegant way to demonstrate the importance of the non-identifiability in this system. Please see figure 5 in the revised manuscript. We thank the reviewer for their suggestion.
