# Supplementary material for: System identifiability in a time-evolving agent-based model
Source: PLoS One. 2024 Jan 25;19(1):e0290821. doi: 10.1371/journal.pone.0290821 (PMC10810497; doi:10.1371/journal.pone.0290821)
Supplement: Attachment — Submitted filename: Reply to reviewers 2 _ 05.07.2023.docx [file pone.0290821.s004.docx]

Dear Editor,

Below are the comments from reviewers and our point-by-point responses. We hope our response and revision satisfies all concerns and will be happy to make additional changes if those are deemed necessary.

We addressed all editorial comments in the submission and made a depository of our data and the algorithms that produce all figures:

<https://github.com/TalRobin/ABM_Identifiability>

All the best,

Tal Robin.

## Reviewer comments:

### Reviewer 1:

I still think the paper has value for the scientific community, but I regret to inform you that the previous issues have not been satisfactorily addressed. Moreover, I have failed to properly carry out this revision due to the problems I will discuss below.

Reply: We hope this new round of revisions satisfies your concerns.

1. Saying “We therefore propose a likelihood-based approach to discern whether model parameters have been properly identified” still gives me the feeling that a general procedure will be described, while the paper is a particular application. Moreover, the cited lines in the response did not match the accessible material.

Reply: While it is true that we described our approach in the narrow application of our model, it was done as an example of the utility of using synthetic trajectories for examining the identifiability of a system. We have rephrased the sentence to avoid misrepresentation:

“show that analyzing synthetic trajectories can support or contradict claims of identifiability. While we perform this on a specific model system, this approach can be generalized for a variety of stochastic representations of partially observable systems.”

(See line 27 in the revised manuscript)

2. In this version a couple of the references appear to have errors and can not be properly seen.

Reply: We thank the reviewer for bringing this to our attention. We have reentered all references and checked that the doi numbers refer to the correct papers. We hope this is now resolved.

3. The previous issue 4. (The term "demonstrated" might lead to the thought that a demonstration has been developed rather than an interpretation of results) is not actually addressed.

Reply: In our initial response, we understood your remark as an objection to the extent to which a demonstration is performed and therefore tried to soften it. However, we now see that we misunderstood your meaning, and that your objection is to our claim that non-identifiability in this system is caused by an interplay between parameters, which is mere interpretation of the results. We agree that this is not a fact in the strictest sense, and therefore adjusted the text appropriately :

“Our findings can be reasonably interpreted as an example of this issue. The linear dependance between the inferred parameters along the ridge indicates that an interplay between the parameters may be the cause of unidentifiability.”

(See line 310 in the revised text)

4. There exists at least a few differences between the version with the track of changes and the ones without. The aforementioned problems make it so time-consuming to properly revise the paper that a could just make a partial revision which made me suggest a major revision.

Reply: We apologize for the confusion. When we rearranged the location of the methods and some of the figures while track-changes was on, the Word file crashed. Therefore, we had to make these changes without tracking changes. The newly submitted file is tracked properly from the last submission and this should no longer be an issue.

### Reviewer 2:

I appreciate the effort made by the authors to address my comments. However, I think most of my comments were either avoided or ignored. Overall, the manuscript can benefit of clear references of previous studies to back up their claims, as well as improvement on their experiments' design and description.

Reply: Our apologies for this impression. Our intention was not to ignore or avoid your comments. We hope all comments are now properly addressed.

1. In my first comment, I mentioned that the authors already outlined some references for methods developed to improve parameter identifiability, but there isn't a clear argument of the reason to not use those methods in this manuscript.

Reply: Our intention was to include a more robust assessment of these methods in a separate publication. However, to comply with the reviewer’s request, we include in the revised manuscript initial results from implementing observation segmentation. As can be seen in new Figure 7 and the corresponding text (lines 286-298 in the revised manuscript), using this method can produce some improvement in identifiability. However, it is important to state that this improvement is only exemplified, and the systematic advantage needs more extensive examination.

2. I also don't think comment 3 was properly addressed. The authors added a figure (Figure 5) in the manuscript but did not elaborate on the rationality for this experiment in the methods section. What were the authors expected to observe? Why this specific scenario?

Reply: We thank the reviewer for insisting on these important points. Our rational for this experiment is to show that even though observed time series of incidence are indistinguishable under different parameters, outcomes of infection and colonization might differ once the system is perturbed by an intervention. We used a very simple representation to do so and chose a rather strong intervention to emphasize the point. We have adjusted the description of Figure 5 (lines 269-275 in the revised manuscript) in a way that we hope will clarify this issue.

3. Similarly, the authors mentioned that they " we prefer to avoid pointing fingers to any specific publications and hope that people will take this message to heart regardless." But I don't understand. How are the readers supposed to know that this is a real issue if it's not exemplified with references. I personally think that the way to illustrate an issue in a scientific manuscript is to provide references and not to expect readers to take the message to heart without evidence.

Reply: While we are certain that the issue of too much trust being given to inferenced models without proper examination of identifiability is a widespread phenomenon and should be taken to consideration more often, we feel that it is not only unwise, but also unfair, to name specific people who fell to this practice which is so widespread. However, it is a fair point that when one is making a claim one must support it. Therefore, we are changing the claim that parameter identifiability is often not addressed to:

“the problem of non-identifiability in a stochastic system which, when overlooked, can significantly impede model prediction” in the abstract (line 19 in the revised text), and in the introduction to:

“the issue of parameter identifiability, which, if not addressed properly can distort prediction and system certainty” (line 59 in the revised text) and provide two references to support this claim.

4. This issue comes up again in my comment #12. The authors added a whole new experiment, but did not describe this experiment in the methods.

Reply: A section in the methods describing the experiment is now added (lines 175-196 in the revised text).
